# The establishment of dynamic microexpression training tool

**DOI:** 10.3389/fpsyg.2025.1676880

**Published:** 2026-01-29

**Authors:** Jianxin Zhang, Ning Cai, Lihan Xu, Jinghua Liu, Yichen Li, Xiaodan Wang, Ming Yin

**Affiliations:** 1 Laboratory of Learning and Teaching Brain Science, School of Education, Jiangnan University, Wuxi, China; 2 Jiangsu Province Engineering Research Center of Microexpression Intelligent Sensing and Security Prevention and Control, Nanjing, China; 3 Jiangsu Micro Digital Technology Co., Ltd., Nanjing, China; 4 Department of Investigation, Jiangsu Police Institute, Nanjing, China

**Keywords:** dynamic microexpression, DMETT, recognition technique, training effect, gender difference

## Abstract

The existing microexpression training tool METT only trains the static microexpression recognition ability under neutral background, but does not train the dynamic microexpression recognition ability under expression backgrounds. Therefore, in the current study, the dynamic microexpression recognition ability test DMERT was used as pretest and posttest, the METT recognition techniques were guided and trained, and the dynamic microexpression training tool DMETT was established as a training tool for dynamic microexpression recognition ability. The experiment was conducted in 3 stages (pretest vs. training vs. posttest) × 7 (background expression: sadness vs. Disgust vs. fear vs. anger vs. surprise vs. happiness vs. Neutral) × 2 (background expression emotional arousal: 3 vs. 5) × 6 (dynamic microexpression: sadness vs. Disgust vs. fear vs. anger vs. surprise vs. happiness) × 2 (emotional arousal of dynamic microexpression: 1 → 2 → 3 → 2 → 1 vs. 3 → 4 → 5 → 4 → 3). The results showed that: (1) The DMETT could effectively improve dynamic microexpression recognition ability with good reliability and validity. (2) The METT recognition techniques were suitable for training dynamic microexpression recognition ability. (3) The DMETT could be used as a training tool of dynamic microexpression recognition ability, and can be further verified to serve as a measurement tool of microexpression learning ability in the future. (4) There was no gender difference in general dynamic microexpression recognition ability, but females outperformed males in general dynamic microexpression recognition learning with small effect sizes.

## 1 Introduction

Microexpression is a particularly short expression of about 1/25 ~ 1/2 s. It is difficult to control so that it can reveal the real emotions that people try to suppress or hide as an effective way to detect lie (Shen et al., 2017). The microexpression recognition ability refers to the ability of people to recognize the microexpressions of others. It is an important part of emotional intelligence to detect the true emotions of others and carry out appropriate social communication. Existing studies have established an operational definition of microexpression by taking static transient expression as an approximation of microexpression embedded in a neutral background or an expression background. They developed JACBART (Japanese and Caucasian Brief Affect Recognition Test, Matsumoto et al., 2000), EMERT (Ecological Microexpression Recognizition Test, Zhang et al., 2017; Yin et al., 2020; Zhu et al., 2017; Zhu et al., 2019), WEMERT (Weak EMERT, Yin et al., 2019), and PREMERT (Pseudorandom EMERT, Zhang et al., 2020, 2022) to investigate people’s microexpression recognition ability. Zhang et al. (2022) extracted common factors from PREMERT by using exploratory factor analysis to propose the general microexpression recognition ability. Zhang et al. (2024) used several transient static expressions with gradual emotional arousal with both weak and strong peaks under expression backgrounds as the approximation of dynamic microexpressions to establish the dynamic microexpression recognition ability test DMERT.

It is easy for people to recognize expressions, but it is very difficult to recognize microexpressions. Without training, the correct rate is 0.45–0.59 (Matsumoto and Hwang, 2011). Therefore, training is needed to improve the microexpression recognition ability, so as to detect the true emotions of others in a more timely and accurate manner, handle interpersonal relationships, and improve social communication ability and mental health level. Ekman (2002, 2003) established a classical microexpression recognition training tool METT to train seven basic microexpression recognition abilities, and JACBART test was used in the pre and post test stage. In the training stage, participants were asked to watch videos explaining the characteristics and recognition techniques of microexpression, and practiced the recognition techniques. It was found that METT could improve the recognition ability of microexpression in various groups of people, such as university students, department store employees, interrogation consultants, coast guard members, police officers, customs officers, and lie detectors (Cao, 2011; Ekman, 2009; Endres and Laidlaw, 2009; Frank et al., 2009; Frank and Svetieva, 2015; Hall and Matsumoto, 2004; Hurley, 2012; Hurley et al., 2014; Matsumoto and Hwang, 2011; Warren et al., 2009; Wu et al., 2022; ten Brinke et al., 2015). However, METT only trains static microexpression recognition under neutral background. Therefore, Yin et al. (2016) propose to build a tool for training static microexpression recognition under various expression backgrounds.

There are four main behavioral characteristics of real microexpression: (1) dynamic change; (2) different emotional arousal; (3) under various expression backgrounds; and (4) microexpressions and expression backgrounds appear randomly. Most of the above microexpression studies use transient static expression as the approximation of microexpression, without considering the dynamic feature. Yan et al. (2013) found that the real microexpression can be divided into three dynamic stages like expression: emergence → peak → extinction, but the time is shorter. Yan et al. (2013) improved the depression-evoked paradigm to construct CASME; Yan et al. (2014) further constructed CASMEII to provide database support for Chinese localized microexpression researches. They adopted the laboratory depression-evoked paradigm: participants were asked to watch emotional pictures, and the real expressions induced by emotional materials must be suppressed during the whole process, and the neutral expression must be maintained. If repression failed, dynamic microexpressions would be revealed briefly. However, the emotional material has little correlation with participants’ self-expression, and the emotional arousal of dynamic microexpression peak and facial muscle changes are weak, which is different from the real microexpression. The main distinction between macroexpressions and microexpressions is the length of the expressions rather than their intensity (Ekman and Friesen, 1969; Shen et al., 2012; Takalkar et al., 2018). Only Zhang et al. (2024) established the dynamic microexpression recognition ability test DMERT, but they did not train dynamic microexpression recognition ability.

Therefore, in the current study, the dynamic microexpression recognition ability test DMERT was used as pretest and posttest, and the METT techniques were used to guide training methods to improve dynamic microexpression recognition ability. The dynamic microexpression recognition training tool DMETT can be established to improve the dynamic microexpression recognition ability with the reliability and validity tests.

## 2 Materials and methods

### 2.1 Participants

Based on the sample sizes of existing studies on microexpression recognition ability (ranging from 22 to 98 participants, Yan et al., 2013; Yin et al., 2019; Yin et al., 2020; Yin et al., 2020; Zhang et al., 2017; Zhang et al., 2020, 2022; Zhu et al., 2017; Zhu et al., 2019), a total of 104 participants, 16 males and 88 females, were sampled from undergraduates in Jiangnan University in China to participate in the study. In 10 participants, the data in the pretest or posttest stage were lost due to program errors, and the training effect could not be measured; Or they dropped out of the experiment midway and were deleted as invalid data. A total of 94 participants were included in the data analysis, including 16 males and 78 females, aged *M* ± *SD* = 21.41 ± 0.74 years old. All of them were the yellow-skinned people of China, right-handed, with normal corrected vision and no color blindness. The participants had no experience in participating in similar studies. Among them, there were only 89 participants with effective data in the training stage, 5 fewer than the 94 in the pre and post test stage. However, these 5 participants all completed the three stages including the training stage, but the data in the training stage was lost due to a program error, so the data in the pre and post test stage was still valid. All the participants participated voluntarily and could quit at any time. They filled in the informed consent before the experiment and got the corresponding remuneration after the experiment was completed. The experiment followed the ethical guidelines of the 2013 Revision of the Declaration of Helsinki and was approved by the Medical Ethics Committee of Jiangnan University in China. The current study was not preregistered anywhere. The pretest stage used the participants’ data in Zhang et al. (2024), and the participants continued to complete the training stage and the posttest stage in the current study. The current study focused on examining the training effect rather than the pretest dynamic microexpression recognition ability in Zhang et al. (2024), which was the difference and innovation.

### 2.2 Experimental instruments and materials

Facial Expressions of Emotion – Stimuli and Tests (FEEST) by Young et al. (2002) based on Ekman and Friesen (1976), half male and half female, with a total of seven expression types, namely sadness, fear, disgust, anger, surprise, happiness and neutral. The emotional arousal of the first six expression pictures was 1–5, and the emotional arousal of neutral was 0. The pictures were processed to remove other parts except facial muscles, such as ears and hair, etc., so as to ensure the same person’s facial expression with the same shadow and head posture. All images were altered to 338 pixels by 434 pixels with a grey background (GRB: 127, 127, 127).

The experimental instrument was Lenovo laptop Legion Y7000 2019 PG0, with the 15.6 inches display, 1920 × 1,080 resolution, 60 Hz refresh frequency, gray display background, and the standard keyboard as the experimental reaction instrument. The experimental program was prepared by E-prime 3.0.

### 2.3 Experimental design and procedures

The current study improved the experimental paradigm of METT (Ekman, 2002, 2003), and replaced the static short expression picture with 5 short static expression pictures with gradual emotional arousal to approximate the dynamic microexpression as DMERT (Zhang et al., 2024). The experiment consisted of 3 (stage: pretest stage vs. training stage vs. posttest stage) × 7 (background expression: sadness vs. disgust vs. fear vs. anger vs. surprise vs. happiness vs. neutral) × 2 (background expression emotional arousal: 3 vs. 5) × 6 (dynamic microexpression: sadness vs. disgust vs. fear vs. anger vs. surprise vs. happiness) × 2 (emotional arousal of dynamic microexpression: 1 → 2 → 3 → 2 → 1 vs. 3 → 4 → 5 → 4 → 3) × 2 (group: group A vs. group B) mixed experimental design. The first 5 independent variables were intra-subject variables; The last independent variable was the inter-subject variable.

The purpose of this design was: (1) The pretest stage measured the baseline level of dynamic microexpression recognition ability of participants without training; The training stage was to learn and practice the dynamic microexpression recognition techniques; The posttest stage measured the level of dynamic microexpression recognition ability of the trained participants. Then the difference in accuracy among the three stages was the training effect. (2) Of the 7 background expressions, except for neutral, 6 background expressions had two kinds of emotional arousal, weak and strong, which was to ensure that the background expression had a good ecological validity; 6 kinds of dynamic microexpressions had two kinds of emotional arousal, weak and strong, which was also to ensure that dynamic microexpressions had good ecological validity. The six dynamic microexpressions were embedded in the seven background expressions, and the emotional titer effect and background effect of the dynamic microexpressions were investigated. (3) One white male model and one white female model were used in each stage. In order to reduce the practice effect, different models were used in the three stages. In order to balance the influence of facial preference, group A and group B were set up in the pretest stage and posttest stage, and the participants were divided half and half. The pretest stage of group A was taken as the posttest stage of group B.

**Figure 1:**
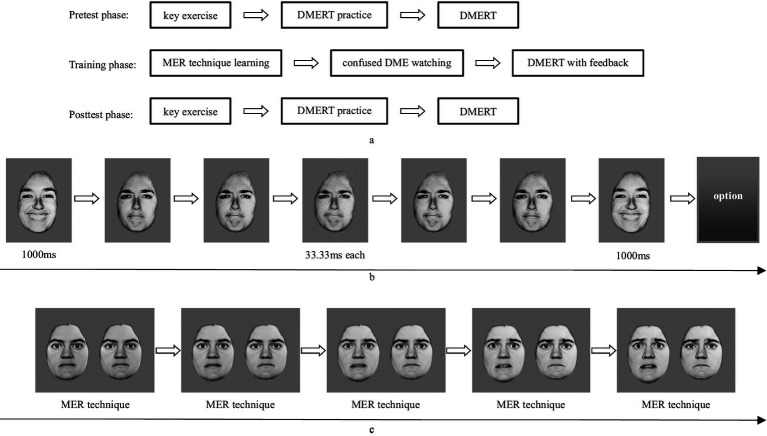
**(a)** Flow chart of DMETT. **(b)** Flow chart of dynamic microexpression recognition for a single trial. Adapted with permission from copyright holder Young et al. (2002). **(c)** Flow chart of confused DME watching (fear and sadness).

The construction of a single trial of dynamic microexpression in the three stages was the same: a certain expression picture of a model, happiness for example, was selected as the background expression before and after, and the presentation time was 1,000 ms; five short expression pictures, sadness for example, with the gradual emotional arousal of the model, 1 → 2 → 3 → 2 → 1 for example, were selected as the dynamic microexpression, and the presentation time was 33.33 ms each, and the five images were 167 ms in total (see Figure 1b). Since there were 7 background expressions and 6 dynamic microexpressions, the pseudo random design was used to balance the sequence effect.

Each participant was required to complete the three stages three times within 72 h (3 days), and the interval between the two stages was at least 3 h. The purpose was to ensure that the participants had enough time for automatic learning in brain cognitive processing, and to balance the effect of practice, fatigue and forgetting. Each stage was completed on a computer, with the participants’ eyes 60 cm away from the screen.

Pretest stage: Before the experiment began, the participants were asked to place their left ring finger, middle finger and index finger on the S, D and F keys respectively, and their right index finger, middle finger and ring finger on the J, K and L keys respectively, corresponding to sadness, disgust, fear, anger, surprise and happiness. Then, the key exercise was carried out. Six expression words (except neutral) appeared pseudo-randomly twice in 1 s. The participants were required to select the right expression word by pressing the key on the option page that presented the six expressions in order. After the keystroke practice was completed, the experimental instruction was presented to inform the participants: “Please look at the center of the screen carefully, you will see a fixation point + on the screen, after that, you will see the first expression picture for 1 s, and then the microexpression pictures will flash quickly, that is, there are five microexpression pictures, all of which are the same microexpression type, but their emotional arousal is gradually changing, so there will be a feeling of movement. And then the first expression picture will appear again for 1 s. Please select the microexpression type you see, only as accurate as possible, not as fast as possible. After the option is selected, the screen is empty for 1 s, and then the fixation point + is displayed for 500 ms, and the next trial begins.” The process of dynamic microexpression recognition for a single trial is shown in Figure 1.

First, the dynamic microexpression recognition program was practiced for a total of 14 trials, in which 7 background expressions appeared twice and 6 microexpressions appeared 2–3 times. Then the formal experiment was conducted. In order to allow the participants to have a full rest, the experiment was divided into 6 blocks with 52 trials in each block, and a total of 312 trials were conducted, including [6 (background expression: sadness vs. disgust vs. fear vs. anger vs. surprise vs. happiness) × 2 (background expression emotional arousal: 3 vs. 5) + 1 (background expression: neutral) × 1 (emotional arousal of background expression: 0)] × 6 (dynamic microexpression: sadness vs. disgust vs. Fear vs. anger vs. surprise vs. happiness) × 2 (emotional arousal of dynamic microexpression: 1 → 2 → 3 → 2 → 1 vs. 3 → 4 → 5 → 4 → 3) × 2 (Model: Male vs. Female) = 312 trials. The participants need rest 60 s between two blocks.

Training stage: Firstly, the participants learned the recognition techniques of two easily confused dynamic microexpressions from METT in written form on the computer screen (Ekman, 2002, 2003, see the next paragraph). When the participants felt that they had mastered the content, they pressed the button to end the text display and start observing the pictures. Then, to show the different details, the two easily confused dynamic microexpressions were presented slowly in pair for 500 ms, for example, the fear picture was on the left, while the sadness picture was on the right. The emotional arousal of the pair changed in the order of 1 → 2 → 3 → 4 → 5 to create a dynamic change, and the corresponding recognition techniques were marked with text below the expressions to deepen the experience and learning of recognition techniques. The cycle of emotional arousal changes occurs 12 times, resulting in 5 × 12 = 60 pairs and 0.5 s/pair × 60 pairs = 30 s. There were four types of two easily confused dynamic microexpressions, such as fear and sadness, fear and surprise, anger and disgust, and happiness and neutral.

The recognition techniques are as follows: (1) Fear and sadness: Fear and sadness are often confused, both eyebrows are raised. But fear’s eyebrow is balanced and raised straight up, while sadness’s inner brow is raised. The lips of fear are extended horizontally. The sad jaw is lifted slightly, while the corners of the mouth are slightly lowered. (2) Fear and surprise: Fear and surprise are often confused. Both eyebrows are raised. But the eyebrows of fear are not only raised, they also straighten and converge; The eyebrows of surprise are curved. The eyes of fear have more glare in them than the eyes of surprise. Although the lips are all open, the lips of fear are more tense than the lips of surprise. (3) Anger and disgust: Anger and disgust are sometimes confused, with both eyebrows hanging down. But the eyes of anger are glared. The eyes of disgust are narrowed rather than glared. Look at how tight the angry lips on the left are and how relaxed the disgust lips on the right are. All the movements of disgust are centered around the center line. (4) Happiness and neutral: The corners of the mouth are raised and the eyes are squinted when happiness.

After that, the participants were asked to use the learned recognition techniques to recognize dynamic microexpressions of different models from pretest phase, and the results of correct recognition were feedback, and the peak pictures of correct dynamic microexpressions were presented to further deepen the learning. There were 6 blocks in the stage, each had 52 attempts, a total of 312 trials (same as the pretest stage).

Posttest phase: same as pretest phase, only with different models.

The experimental procedures were shown in Figure 1.

### 2.4 Steps of data analysis

Spss 19.0 was used for statistics. Draw on existing studies (Ekman, 2002, 2003; Yin et al., 2020; Zhang et al., 2022; Zhang et al., 2024), data analysis consists of four steps:

1. Calculate the effectiveness of DMETT

Alternate form test: Since the pretest of group A was the posttest of group B, and the posttest of group A was the pretest of group B, the tests of the same model were combined, that is, the pretest of group A and the posttest of group B were combined to form the model X group, and the posttest of group A and the pretest of group B were combined to form the model Y group. The recognition accuracy of model X and Y groups was compared by ANOVA to test whether there was difference in pretest and posttest in both group A and group B.

ANOVA: With dynamic microexpression recognition accuracy as the dependent variable, repeated measurement ANOVA of 3 (stage) × 7 (background expression) × 6 (dynamic microexpression) was performed. The training effect of DMETT can be obtained by calculating the main effect of the stage and its interaction effect. The emotional arousal of background expression and microexpression was not investigated, because if the two were investigated, the trial number of each level of independent variable would be too small. However, if they were combined to calculate the accuracy, these two independent variables were included, which guaranteed the ecological validity. The independent variables of the group were not considered, in order to balance the influence of the model’s face preference as much as possible and increase the face diversity.

2. Quantifying the training effect of DMETT.

Dynamic microexpression recognition ability has three layers. The first layer is 42 subcategories of dynamic microexpressions, namely 6 dynamic microexpressions under 7 background expressions. The second layer is 6 categories of dynamic microexpressions, that is, the average recognition accuracy of a certain type of dynamic microexpression under seven background expressions (such as sad dynamic microexpression under the backgrounds of sadness, disgust, fear, anger, surprise, happiness and neutral) is taken as the recognition accuracy of this category of dynamic microexpression, and the standard deviation is taken as the background effect of this category of dynamic microexpression. The third layer is the general dynamic microexpression. The average of the six categories of dynamic microexpression (namely, the sum of the recognition accuracy of the six categories of sadness, disgust, fear, anger, surprise and happiness is divided by 6) is taken as the general dynamic microexpression recognition ability MM, and the standard deviation is the emotional titer effect of the general dynamic microexpression recognition ability MS. The average of the background effects of 6 categories of dynamic microexpressions as the background effect of general dynamic microexpressions SM, and the standard deviation is the emotional titer effect of general dynamic microexpression background effect SS (that is, the difference degree of background effects of different microexpressions). The accuracy of 42 dynamic microexpressions and 6 categories of dynamic microexpressions were compared with the random level 1/6 (6 options per trial) by single-sample *t-*test to investigate which was higher than random; The general dynamic microexpression recognition accuracy was compared with random level 1/6 and 0.5 (right or wrong) by single sample *t-*test to investigate general dynamic microexpression recognition ability level. The background effect and emotion titer effect were compared with 0 by single sample *t-*test to investigate the recognition stability.

Quantitative indexes of DMETT training effect: For 42 dynamic microexpressions, 6 categories of dynamic microexpressions and general dynamic microexpressions recognition accuracy and background effect, three difference values were obtained by subtracting the pretest stage from the training stage, subtracting the training stage from the posttest stage, and subtracting the pretest stage from the posttest stage as the quantitative indexes of the training effect. The single sample *t* test was performed with the random level 0 to get which training effects were higher than random.

The principle of multiple comparison correction is as follows: Suppose that there were 10 multiple comparisons among 5 variables, to ensure that the entire *p* value is less than 0.05, each comparison *p* value should be less than 0.005 (0.05÷10 = 0.005, Holm, 1979). However, in the current study, each dynamic microexpression was compared with 1/6 or 0.5 only once without compering with the other dynamic microexpressions, and each training effect was compared with 0 only once without compering with the other training effects. Since no multiple comparison occurred, it was neither need nor possible to perform multiple comparison correction in the current study. For detailed explanations, please refer to the existing researches (Yin et al., 2019; Yin et al., 2020; Zhang et al., 2017; Zhang et al., 2024; Zhang and Liu, 2021; Zhang et al., 2020, 2022).

3. Reliability and validity tests for DMETT.

The split-half reliability test was adopted. The training effect was divided into odd and even trials, and then the Pearson correlation analysis was conducted on the odd and even training effects. The criterion-related validity test was adopted. In DMETT, the training of dynamic microexpressions under neutral background was an approximation of METT. Therefore, the METT training effect with neutral background as the criterion was used, and the Pearson correlation analysis was performed between DMETT and METT training effects.

4. Gender difference of DMETT.

The one-way ANOVA was used to explore gender difference of DMETT with the general microexpression or its training effect as dependent variable, and gender as the independent variable.

The effect size (Cohen’s *d* or η^2^) is too small below 0.20, small from 0.20 to 0.50, medium from 0.50 to 0.80, and big above 0.80 (Cohen, 1988).

## 3 Results

### 3.1 Effectiveness of DMETT

**Figure 2:**
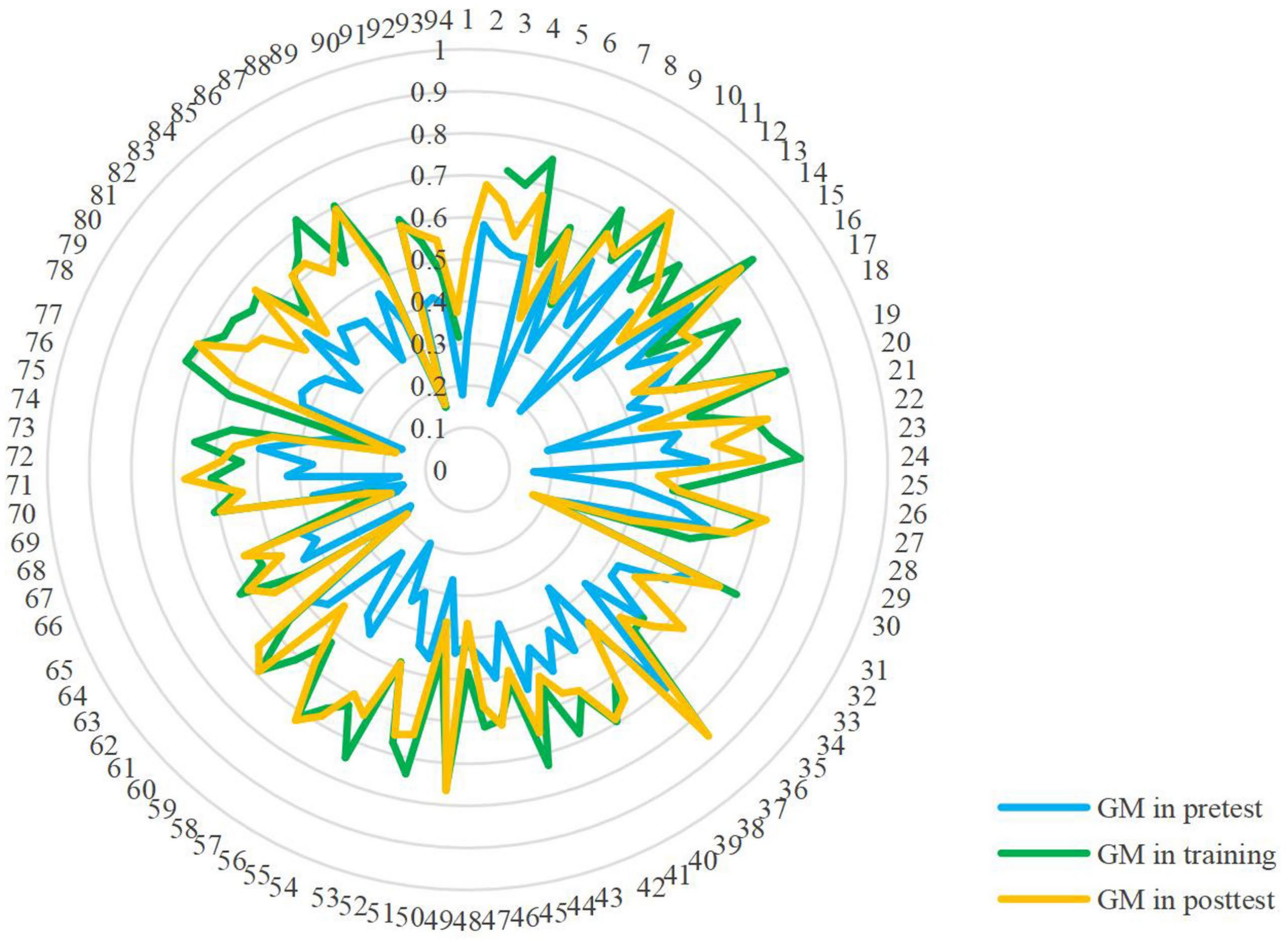
Scores of general microexpression (GM) in three stages. The numbers on the outermost circle represented the participant numbers.

The accuracy of dynamic microexpression recognition in 42 subcategories, the accuracy and standard deviation of dynamic microexpression recognition in 6 categories, the accuracy of general dynamic microexpression recognition, background effect and the single sample *t-*test results were shown in Table 1, Figure 2, and Appendix Table 7. The accuracy of general dynamic microexpression recognition MM of model X group and Y group was compared by ANOVA. The X group was *M* ± *SD* = 0.49 ± 0.15, the Y group was *M* ± *SD* = 0.47 ± 0.16, and the difference was not significant, *F* (1, 186) = 0.95, *p > 0.*05, η^2^ = 0.005. The result showed that pretest and posttest in both group A and group B were indeed alternate forms of each other.

**Table 1:** Scores of dynamic microexpression in three stages.

Dynamic microexpression	Pretest phase (*M* ± *SD*)	Single sample *t*-values	Cohen’s *d*	Training phase (*M* ± *SD*)	Single sample *t*-values	Cohen’s *d*	Posttest phase (*M* ± *SD*)	Single sample *t*-values	Cohen’s *d*
Sadness microexpression	0.28 ± 0.16	6.7***	0.71	0.59 ± 0.19	20.87***	2.23	0.45 ± 0.2	14.14***	1.42
Disgust microexpression	0.42 ± 0.17	14.42***	1.49	0.68 ± 0.18	26.86***	2.85	0.64 ± 0.2	22.68***	2.37
Fear microexpression	0.24 ± 0.15	4.67***	0.49	0.4 ± 0.17	12.8***	1.37	0.39 ± 0.19	11.14***	1.18
Anger microexpression	0.23 ± 0.13	4.88***	0.49	0.37 ± 0.15	12.54***	1.36	0.34 ± 0.22	7.31***	0.79
Surprise microexpression	0.62 ± 0.22	19.47***	2.06	0.67 ± 0.17	28.53***	2.96	0.71 ± 0.19	27.18***	2.86
Happiness microexpression	0.66 ± 0.24	19.65***	2.06	0.85 ± 0.18	35.38***	3.8	0.83 ± 0.18	35.83***	3.69
Sadness background effect	0.22 ± 0.08	27.15***	2.75	0.21 ± 0.06	33.17***	3.5	0.24 ± 0.07	35.05***	3.43
Disgust background effect	0.21 ± 0.07	27.05***	3	0.16 ± 0.06	26.97***	2.67	0.19 ± 0.06	30.75***	3.17
Fear background effect	0.18 ± 0.07	26.8***	2.57	0.23 ± 0.07	31.93***	3.29	0.2 ± 0.07	29.06***	2.86
Anger background effect	0.19 ± 0.08	22.65***	2.38	0.17 ± 0.05	30.59***	3.4	0.17 ± 0.07	22.4***	2.43
Surprise background effect	0.24 ± 0.07	30.96***	3.43	0.21 ± 0.06	33.44***	3.5	0.18 ± 0.07	26.07***	2.57
Happiness background effect	0.26 ± 0.08	30.5***	3.25	0.12 ± 0.06	18.55***	2	0.13 ± 0.07	18.78***	1.86
General microexpression MM	0.41 ± 0.13	7.05***	−0.69	0.59 ± 0.14	6.2***	0.64	0.56 ± 0.14	3.95***	0.43
General microexpression MS	0.22 ± 0.09	25.03***	2.44	0.21 ± 0.06	32.08***	3.5	0.24 ± 0.07	33.7***	3.43
General microexpression SM	0.21 ± 0.04	46.36***	5.25	0.18 ± 0.03	58.81***	6	0.19 ± 0.03	53.87***	6.33
General microexpression SS	0.07 ± 0.02	28.67***	3.5	0.07 ± 0.02	34.90***	3.5	0.07 ± 0.02	34.22***	3.5
Classic microexpression	0.46 ± 0.17	−2.24*	−0.24	0.64 ± 0.15	8.74***	0.93	0.59 ± 0.17	5.08***	0.53
Classic microexpression expression effect	0.35 ± 0.09	39.19***	3.89	0.31 ± 0.08	35.92***	3.88	0.34 ± 0.09	38.94***	3.78

*n* = 94 for pretest and posttest, *n* = 89 for training. Single sample *t-*value, for 6 categories of dynamic microexpression recognition accuracy, the comparison standard was 1/6; For background effect and expression effect, the comparison standard was 0; For general microexpression MM and classic microexpression, the comparison standard was 0.5. * Means *p* < 0.05, ** means *p* < 0.01, and *** means *p* < 0.001, the same was as below.

With dynamic microexpression recognition accuracy as the dependent variable, repeated measurement ANOVA of 3 (stage) × 7 (background expression) × 6 (dynamic microexpression) was performed:

1. The sphericity test of the stage was significant, *p <* 0.05. After Greenhouse correction, the main effect of the stage was significant, *F*(1.71, 89) = 197.44, *p* < 0.001, η^2^ = 0.692 (medium), indicating that the stage affected the dynamic microexpression recognition ability. The paired comparison showed that the pretest stage was smaller than the training stage and the posttest stage, and the training stage was larger than the posttest stage, *p*s<0.001. The pretest stage was greater than 1/6 but less than 0.5, and the training stage and the posttest stage were both greater than 0.5 (see Table 1). These indicate that there was a significant training effect.
2. The sphericity test of the background expression was significant, *p < 0.*05. After Greenhouse correction, the main effect of the background expression was significant, *F*(5.*2*4, 89) = 45.34, *p* < 0.001, η^2^ = 0.340 (medium), indicating that the background expression affected the dynamic microexpression recognition ability.
3. The sphericity test of dynamic microexpression was significant, *p* < 0.05. After Greenhouse correction, the main effect of microexpression was significant, *F*(3.40, 89) = 338.70, *p* < 0.001, η^2^ = 0.794 (medium), indicating that the type of dynamic microexpression affected the dynamic microexpression recognition ability.
4. The sphericity test of dynamic microexpression × background expression × stage was significant, *p <* 0.05. After Greenhouse correction, interaction effect among the three was significant, *F*(15.11, 89) = 9.26, *p* < 0.001, η^2^ = 0.095 (too small). A further analysis on the interaction effect of ANOVA would be too complex to understand, the effect size was too small so that there was little value to analyze the interaction effect, and the focus of the current study was to examine the training effect. Therefore, in the following section 3.2, the training effect was quantified and statistically analyzed as an alternative solution (Yin et al., 2020). Because the quantitative training effect analysis contained comparisons of different stages, dynamic microexpressions and backgrounds, and some interaction effects of them (see section 3.2 and Table 2).

**Table 2:** Quantitative indicators of training effect of dynamic microexpressions.

Dynamic microexpression	TPr (*M* ± *SD*)	Single sample *t*-values	Cohen’s *d*	PoT (*M* ± *SD*)	Single sample *t* -values	Cohen’s *d*	PoPr (*M* ± *SD*)	Single sample *t* -values	Cohen’s *d*
Sadness microexpression	0.32 ± 0.17	17.31***	1.88	−0.14 ± 0.15	−8.87***	−0.93	0.17 ± 0.17	9.9***	1
Disgust microexpression	0.27 ± 0.18	14***	1.5	−0.04 ± 0.17	−2.22 *	−0.24	0.23 ± 0.21	10.36***	1.1
Fear microexpression	0.17 ± 0.15	10.41***	1.13	−0.01 ± 0.18	−0.55		0.15 ± 0.22	6.61***	0.68
Anger microexpression	0.14 ± 0.17	7.44***	0.82	−0.03 ± 0.2	−1.45		0.11 ± 0.23	4.4***	0.48
Surprise microexpression	0.06 ± 0.18	3.22**	0.33	0.03 ± 0.15	2.1*	0.2	0.09 ± 0.22	4.03***	0.41
Happiness microexpression	0.2 ± 0.21	9.15***	0.95	−0.03 ± 0.09	−2.94**	−0.33	0.17 ± 0.19	8.7***	0.89
Sadness background effect	−0.01 ± 0.08	−0.96		0.03 ± 0.08	3.3***	0.38	0.02 ± 0.1	1.94	
Disgust background effect	−0.04 ± 0.1	−4.09***	−0.4	0.02 ± 0.07	3.28***	0.29	−0.02 ± 0.09	−1.68	
Fear background effect	0.05 ± 0.09	5.14***	0.56	−0.03 ± 0.09	−3.02**	−0.33	0.02 ± 0.08	2.22*	0.25
Anger background effect	−0.02 ± 0.1	−1.47		0 ± 0.08	−0.15		−0.01 ± 0.13	−1.12	
Surprise background effect	−0.03 ± 0.09	−3.76***	−0.33	−0.02 ± 0.08	−2.64**	−0.25	−0.06 ± 0.09	−5.88***	−0.67
Happiness background effect	−0.14 ± 0.1	−14.26***	−1.4	0.02 ± 0.06	2.67**	0.33	−0.13 ± 0.09	−12.9***	−1.44
General microexpression MM	0.19 ± 0.11	17***	1.73	−0.04 ± 0.07	−4.57***	−0.57	0.15 ± 0.11	13.81***	1.36
General microexpression MS	−0.01 ± 0.09	−1.53		0.03 ± 0.06	4.47***	0.5	0.01 ± 0.09	1.54	
General microexpression SM	−0.03 ± 0.05	−6.09***	−0.6	0 ± 0.04	0.79		−0.03 ± 0.06	−4.94***	−0.5
General microexpression SS	0 ± 0.03	−0.82		0 ± 0.02	1.31		0 ± 0.03	0.06	
Classic microexpression	0.18 ± 0.15	11.94***	1.2	−0.05 ± 0.13	−3.98***	−0.38	0.13 ± 0.15	8.13***	0.87
Classic microexpression expression effect	0.32 ± 0.1	30.52***	3.2	0.27 ± 0.1	25.64***	2.7	0.32 ± 0.12	26.05***	2.67

Pretest and posttest stages were one-to-one correspondences *n* = 94, training stage *n* = 89. TPr was the training phase minus the pretesting phase, so *n* = 89; PoT was the posttest phase minus the training phase, so *n* = 89; PoPr was posttest minus pretest, so *n* = 94. In the t value of a single sample, the comparison standard was 0.

The single sample *t*-test found (see Table 1): (1) In the pretest stage, except for sadness under disgust, fear under disgust, fear under anger, anger under happiness, and fear under neutral, the recognition accuracy of the other 37 subcategories and 6 categories of dynamic microexpression was greater than 1/6; General microexpression MM was greater than the random level of 1/6, *t* (1, 94) = 17.97, *p* < 0.001, *d* = 1.87 (big), but was less than 0.5. Those indicate that without training, the participants initially had a certain degree of dynamic microexpression recognition ability, but it was low. The background effects of 6 categories of dynamic microexpression and general microexpression SM were all greater than 0, indicating the existence of background effect. (2) In the training stage, except for fear under surprise, the recognition accuracy of other 41 subcategories and 6 categories of dynamic microexpressions was greater than 1/6; The general microexpression MM was greater than 0.5. Those indicate that after training, the dynamic microexpression recognition ability of the participants was improved to a higher level. The background effects of 6 categories of dynamic microexpression and general microexpression SM were all greater than 0, indicating the existence of background effect. (3) In the posttest stage, the recognition accuracy of 42 subcategories and 6 categories of dynamic microexpressions was greater than 1/6; The general microexpression MM was greater than 0.5. Those indicate that after training, the dynamic microexpression recognition ability of the participants was improved to a higher level. In the posttest stage, the background effect of 6 categories of dynamic microexpression and general microexpression SM were all greater than 0, indicating the existence of background effect. (4) A few effect sizes (Cohen’s *d*) were small, while the majority were medium or big.

### 3.2 Quantification of DMETT training effects

**Figure 3:**
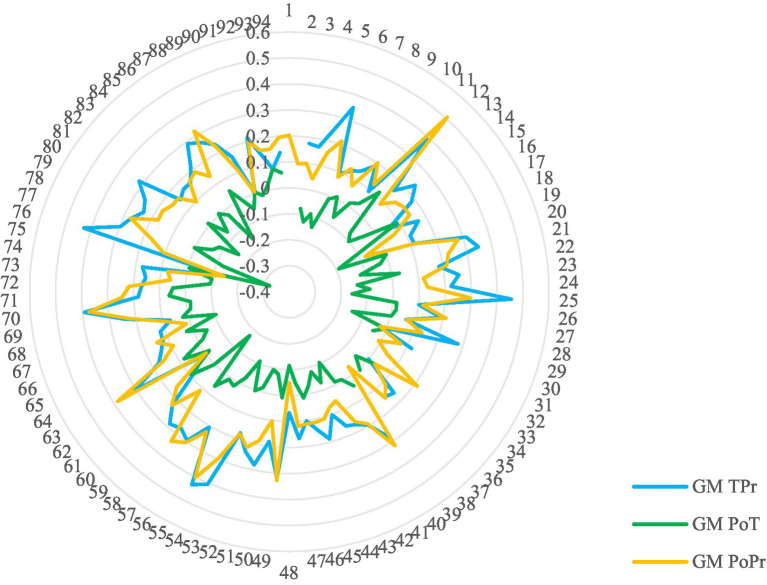
Scores of general microexpression (GM) training. The numbers on the outermost circle represented the participant numbers.

The quantitative indexes of training effects of 42 subcategories of dynamic microexpression, 6 categories of dynamic microexpression and general dynamic microexpression were calculated (see Table 2, Figure 3, and Appendix Table 8). The single sample *t-*test found that: (1) In TPr, except for fear under surprise, anger under disgust, surprise under disgust, fear and anger, the recognition accuracy of other 37 subcategories of dynamic microexpressions, 6 categories of dynamic microexpressions and general microexpressions MM were greater than 0, indicating that compared with the pretest stage, the training stage improved most of the dynamic microexpression recognition ability. However, surprise under anger was less than 0, indicating that the training stage decreased it. The disgust, surprise and happiness background effects, and general microexpression SM were less than 0, indicating that the background effects were reduced by half in the training stage compared with the pretest stage; However, fear background effect was significantly greater than 0, indicating that it was increased in the training stage compared with the pretest stage. (2) In PoT, the recognition accuracy of 13 subcategories of dynamic microexpression, 3 categories of dynamic microexpression and general microexpression MM were all less than 0, indicating that part of dynamic microexpression recognition ability was reduced in the posttest stage compared with the training stage; However, there were 4 subcategories and 1 category of dynamic microexpressions greater than 0, indicating that compared with the training stage, the posttesting stage improved a small part of dynamic microexpression recognition ability. The sadness, disgust and happiness background effects were greater than 0, indicating that compared with the training stage, the background effects were increased by half in the posttest stage. However, fear and surprise background effects were less than 0, indicating that they were reduced in the posttesting stage compared with the training stage. (3) In PoPr, except for fear under surprise, anger under surprise and neutral, surprise under disgust and anger, the recognition accuracy of other 37 subcategories, 6 categories of dynamic microexpressions and general microexpression MM were greater than 0, indicating that compared with the pretest stage, the posttest stage improved most of the dynamic microexpression recognition ability. The surprise and happiness background effects and general microexpression SM were less than 0, indicating that some background effects were reduced in the posttest stage compared with the pretest stage. However, the fear background effect was greater than 0, indicating that it was increased in the posttest stage compared with the pretest stage. (4) A few effect sizes (Cohen’s *d*) were small, while the majority were medium or big.

### 3.3 Reliability and validity of DMETT

Pearson correlation analysis was conducted on the odd and even training effects of PoPr (see Table 3 and Appendix Table 9). Except for disgust under sadness and surprise under sadness, the odd and even training effects of other 40 subcategories of dynamic microexpressions, 6 categories of dynamic microexpressions and general microexpressions MM were correlated, and the odd and even training effects of many background and expression effects were correlated, indicating good split-half reliability for DMETT.

**Table 3:** Correlation between odd and even trials.

PoPr	Odd	Even	*r*
Sadness microexpression	0.16 ± 0.19	0.19 ± 0.19	0.634**
Disgust microexpression	0.23 ± 0.23	0.22 ± 0.23	0.751**
Fear microexpression	0.16 ± 0.23	0.13 ± 0.25	0.676**
Anger microexpression	0.1 ± 0.24	0.11 ± 0.25	0.756**
Surprise microexpression	0.11 ± 0.23	0.07 ± 0.23	0.789**
Happiness microexpression	0.18 ± 0.19	0.16 ± 0.2	0.834**
Sadness background effect	0.33 ± 0.11	0.34 ± 0.11	
Disgust background effect	0.34 ± 0.11	0.34 ± 0.09	
Fear background effect	0.31 ± 0.1	0.33 ± 0.12	
Anger background effect	0.33 ± 0.11	0.33 ± 0.1	0.332**
Surprise background effect	0.33 ± 0.1	0.34 ± 0.12	0.246*
Happiness background effect	0.3 ± 0.11	0.29 ± 0.11	0.574**
General microexpression MM	0.16 ± 0.12	0.15 ± 0.11	0.826**
General microexpression MS	0.19 ± 0.07	0.21 ± 0.07	0.601**
General microexpression SM	0.32 ± 0.05	0.33 ± 0.05	0.380**
General microexpression SS	0.1 ± 0.03	0.1 ± 0.03	
Classic microexpression	0.14 ± 0.17	0.11 ± 0.17	0.527**
Classic microexpression expression effect	0.4 ± 0.13	0.4 ± 0.15	0.274**

**Figure 4:**
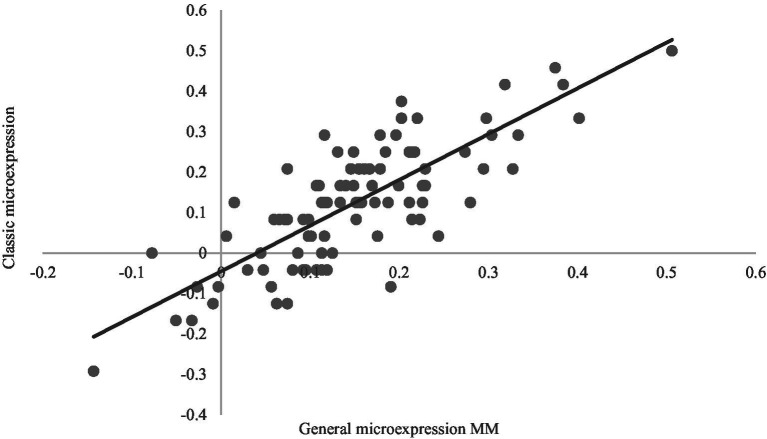
Correlation between PoPr of General microexpression MM and PoPr of classic microexpressions (*r* = 0.797***).

Pearson correlation analysis was performed between DMETT and METT training effects (PoPr, see Tables 4, 5, Figure 4, and Appendix Table 10). Except for sadness under sadness and fear, DMETT and METT training effects of other 40 subcategories of dynamic microexpressions, 6 categories of dynamic microexpressions and general microexpressions MM were correlated, and some background and expression effects of DMETT and METT training effects were correlated, indicating good criterion validity for DMETT.

**Table 4:** Correlation between PoPr of dynamic microexpressions and PoPr of classic microexpressions (*r*).

PoPr	Sadness under neutral	Disgust under neutral	Fear under neutral	Anger under neutral	Surprise under neutral	Happiness under neutral
Sadness microexpression	0.633**					
Disgust microexpression		0.662**				
Fear microexpression			0.759**			
Anger microexpression				0.729**		
Surprise microexpression					0.713**	
Happiness microexpression						0.826**
Sadness background effect	−0.248*					
Disgust background effect						
Fear background effect						
Anger background effect				−0.396**		
Surprise background effect						
Happiness background effect						

**Table 5:** Correlation between PoPr of dynamic general microexpressions and PoPr of classic microexpressions (*r*).

PoPr	Classic microexpression	Classic microexpression expression effect
General microexpression MM	0.797**	
General microexpression MS		0.387**
General microexpression SM		−0.301**
General microexpression SS		

### 3.4 Gender difference of DMETT

The single-sample *t*-test revealed that (see Table 6): (1) General microexpressions of males and females were both less than 0.5 in pretest phase, but general microexpressions of males were not different with 0.5 in training and posttest phases, and general microexpressions of females were more than 0.5 in training and posttest phases, showing training effects for both males and females, but more training effects for females. (2) TPr and PoPr of males and females were both more than 0, but PoT of males were not different with 0, and PoT of females were less than 0, showing training effects for both males and females in training and posttest phases, but more forgetting effect for females in posttest phase. (3) The effect sizes (Cohen’s *d*) were medium or big.

**Table 6:** Gender difference of DMETT.

General microexpression	Male	Single sample *t*-values	Cohen’s *d*	Female	Single sample *t*-values	Cohen’s *d*	*F*	η2
Pretest phase	0.38 ± 0.12	−3.88***	−1	0.41 ± 0.13	−6***	−0.69	0.76	
Training phase	0.51 ± 0.15	0.2		0.61 ± 0.13	7.12***	0.85	7.69**	0.081
Posttest phase	0.48 ± 0.15	−0.57		0.57 ± 0.14	4.82***	0.5	6.34*	0.064
TPr	0.13 ± 0.06	9.2***	2.17	0.21 ± 0.11	16***	1.91	7.68**	0.081
PoT	−0.03 ± 0.06	−1.78		−0.04 ± 0.08	−4.2***	−0.5	0.17	
PoPr	0.1 ± 0.07	5.79***	1.43	0.16 ± 0.11	13.08***	1.45	5.15*	0.053

There were 16 males and 78 females in pretest and posttest phases, and 16 males and 73 females in training phase.

Taking the general microexpression and its training effect as dependent variable, and gender as the independent variable, the one-way ANOVA (see Table 6) found that there was no difference between males and females in pretest phase, but the general microexpression of females was greater than that of males in training and posttest phases, leading to the greater TPr and PoPr of females. But the effect sizes (η2) were too small.

## 4 Discussion

### 4.1 The DMETT had good effectiveness to improve the dynamic microexpression recognition ability

The main distinction between macroexpression and microexpression is the length of the expressions rather than their intensity (Ekman and Friesen, 1969; Shen et al., 2012; Takalkar et al., 2018). Yan et al. (2013), Yan et al. (2013, CASME), and Yan et al. (2014, CASME II) found that real microexpression can be divided into three dynamic stages as macroexpression: appearance → peak → fading, but the time is shorter. According to these researches, in the current study, several transient static expressions with gradual emotional arousal of weak and strong peaks, such as 1 → 2 → 3 → 2 → 1 and 3 → 4 → 5 → 4 → 3, were used to approximate the dynamic three stages of appearance → peak → fading of real microexpression. Each microexpression picture only presented 33.33 ms, and the dynamic microexpression composed of 5 microexpression pictures was 167 ms, which is within the time range of 1/25–1/2 s of the definition of microexpression (Shen et al., 2017). Yan et al. (2013) and Yan et al. (2014) adopted the laboratory repression-evoked paradigm, and the correlation between emotional materials and participants’ self-expression was small, so the peak emotional arousal and facial muscle changes of dynamic microexpression obtained were weak, which was different from the real microexpression. In the current study, 5 transient static expressions with gradual emotional arousal were used as the approximation of dynamic microexpression, and two emotional arousal degrees (weak and strong) of dynamic microexpression peak were set. The results showed that the accuracy of dynamic microexpression recognition in the pretest stage was lower than that of static microexpression recognition in previous studies, indicating that dynamic microexpression recognition was different from static microexpression recognition. Dynamic microexpressions were simulated via sequences of transient static expressions. This was innovative but still different from spontaneous microexpression. However, in CASME and CASMEII, because the emotional material had little correlation with participants’ self-expression, the emotional arousal of dynamic microexpression peak and facial muscle changes were weak. The microexpressions in these two databases were different from the real microexpressions. At the very least, they were only weak real microexpressions. Therefore, the current study used both weak and strong intensity of transient static expression sequences to approximate dynamic microexpression. It was a limited but expedient measure to approximate real dynamic microexpression possibly more than CASME and CASMEII. Following the approach of the current study, the rapid presentation of expression videos can also be used as an approximation of dynamic microexpression. In the future, when the spontaneous microexpression databases contain both the weak (e.g., CASME II, Yan et al., 2014) and the stronger (need to be developed) peak emotional arousal and facial muscle changes, DMETT should use spontaneous dynamic microexpression.

Ekman (2002, 2003) developed METT, a classical training tool for microexpression recognition, and found that METT can improve the microexpression recognition ability among different groups of people (Ekman, 2009; Endres and Laidlaw, 2009; Frank et al., 2009; Frank and Svetieva, 2015; Hall and Matsumoto, 2004; Hurley, 2012; Hurley et al., 2014; Matsumoto and Hwang, 2011; Wu et al., 2022; ten Brinke et al., 2015). But METT trains only static microexpression recognition in a neutral background. Yin et al. (2016) proposed that ecological microexpression recognition training tools should be established under multiple expression backgrounds, but did not consider dynamic characteristics. Zhang et al. (2024) established the dynamic microexpression recognition ability test DMERT, but did not train the ability. Therefore, the current study improved METT paradigm, asked participants to learn and use METT recognition techniques to recognize dynamic microexpressions, and established DMETT training tool for dynamic microexpression recognition. It is found that the microexpression recognition ability in the pretest stage was significantly smaller than those in the training stage and the posttest stage, and the microexpression recognition ability in the training stage was significantly larger than that in the posttest stage, indicating that there was a significant training effect in the training stage. This training effect was not only the practice effect of repeated exposure to dynamic microexpression recognition situations (Hurley, 2012; Matsumoto et al., 2000), but also the training effect of recognition techniques (Ekman, 2002, 2003). Because if there was only practice effect, the posttest stage should have the most practice to make the largest recognition accuracy, but the result was that the recognition accuracy in the training stage with recognition techniques learning was greater than that in the posttest stage without recognition techniques learning. It indicates that METT recognition techniques learning helped to improve the dynamic microexpression recognition ability immediately, but there was some forgetting in the posttest stage. During the training phase, the feedback on the results and the display of the correct microexpression peak pictures might also have deepened learning and improved the accuracy. However, some of these effects might have been partially forgotten during the posttest stage. The influences of these three factors (recognition techniques learning, feedback and display of the correct microexpression peak) on the training effect should be further separated through subtraction or addition experiments. The posttest design was still valuable because the retention of training effects could not be inferred without a posttest. In the future, the delayed posttests (several months or years) should be used to detect the long-term retention of training effects (Hurley, 2012; Matsumoto and Hwang, 2011).

From the quantitative index of training effect, compared with the pretest stage, the training stage and the posttest stage improved most of the dynamic microexpression recognition abilities, reduced some of the background effects, but increased the background effect of fear. However, compared with the training stage, only a few dynamic microexpression recognition abilities were improved in the posttesting stage, but some dynamic microexpression recognition abilities were reduced; and many background effects were increased, except that the background effects of fear and surprise were reduced. On the whole, the training successfully improved and enhanced the dynamic microexpression recognition abilities; However, recognition techniques were partly forgotten after learning. Although the statistical results of the quantitative indexes of the training effect were consistent with those of the variance analysis as a whole, the quantitative indexes of the training effect could intuitively and concisely reveal more specific training effects (Yin et al., 2020).

### 4.2 The DMETT had good reliability and validity

The DMETT had good split-half reliability and good criterion validity. The criterion validity was based on correlations with METT (Ekman, 2002, 2003), which itself only addressed static recognition under a neutral background. METT is an authoritative training tool for microexpression recognition ability, ensuring that the training is focused on microexpression recognition ability. Therefore, using METT as the criterion could test whether the DMETT in the current study trained microexpression recognition ability. In the current study, METT was further improved to train dynamic microexpression under a neutral background. Therefore, METT was an adequate standard for validating DMETT. With good split-half reliability and good criterion validity, DMETT can be used as an effective training tool of dynamic microexpression recognition ability to improve lie detection ability, emotional intelligence, and social communication skills (Matsumoto and Hwang, 2011; Shen et al., 2017; Yin et al., 2016). In the future, it can be further verified to serve as a measurement tool of microexpression learning ability by making sure that the training effect has predictive validity for an external learning ability criterion. Then it can identify which people have a higher ability to learn dynamic microexpression recognition and are more suitable to be trained for working in fields such as investigation, interrogation, customs, security, psychological counseling, and business negotiations. This has educational application significance.

In the current study, compared with the pretest stage, 37 of the 42 subcategories of dynamic microexpressions showed positive training effects in the training stage, which was significantly more than the 13/42 proportion of natural exposure training effect of static strong microexpressions (Zhang et al., 2017). The sample size was 50 (37 + 13), greater than 40. The minimum expected count was 17 *>* 5. *χ*^2^ = 28.46, *p* < 0.001. The 37/42 proportion of dynamic microexpression positive training effect in the training stage in the current study was also significantly more than the 5/42 proportion of natural exposure training effect of static weak microexpressions (Yin et al., 2019). The sample size was 42 (37 + 5), greater than 40. The minimum expected count was 1 < 2.5 < 5. After continuous correction, *χ*^2^ = 45.76, *p* < 0.001. After the first microexpression recognition task completed in the pretest, the second microexpression recognition task was completed both in the training stage of the current study and the posttests of the latter two studies (Zhang et al., 2017; Yin et al., 2019). Therefore, they could compare with each other equally. The posttest stage of the current study was not suitable for comparison with the posttests of the latter two studies. Moreover, each microexpression recognition task in the current study had only 312 trials, while the latter two studies had 420 trials. In the training stage of this study, the number of microexpression recognition trials was 216 less than those in the latter two studies. Thus, the latter two studies should have more practice effects. The same batch of microexpression pictures were used in the pre and post test stages of the latter two studies, and their sequences of presentation were the same in the pre and post test stages, which made that their posttest stages were easier and had greater practice effects than the training stage in the current study. The pretest and training stages in the current study were the alternate form designs of dynamic microexpression pictures of different models, which were more difficult and presented in different order, so the practice effect was excluded as far as possible. Although the static microexpressions in the latter two studies lasted for 133 ms, the dynamic microexpressions in the current study lasted for 167 ms, which was slightly longer. However, the peak of the dynamic microexpressions in the current study only lasted for 33 ms. Moreover, Zhang et al. (2024) proved that the recognition of 167 ms dynamic micro-expressions was significantly more difficult with Lower accuracy than that of 133 ms or shorter static microexpressions. Despite those many unfavorable conditions, the training effect in training stage in the current study was significantly more common, indicating that the learning effect of recognition techniques was much better than the practice effect of natural exposure training (Matsumoto et al., 2000; Hurley, 2012; Hurley et al., 2014; Zhang et al., 2017; Yin et al., 2019), and the METT recognition technique of static microexpression under neutral background was applicable to dynamic microexpression recognition under various background expressions in the current study.

### 4.3 The gender differences of DMETT existed with small effect sizes

Frank et al. (2009) compared the microexpression recognition training effects for different genders, but no differences were found. Hurley et al. (2014) also found no differences between genders. However, Hall and Matsumoto (2004) as well as Mufson and Nowicki (1991) discovered that women typically have a higher accuracy in microexpression recognition, even when age and personality differences are excluded. Yin et al. (2020) found males and females had their own advantages and disadvantages in the microexpression recognition and its natural exposure training effect. The recognition of disgust under sadness and training effect of fear under surprise of females were higher than those of males, but the recognition of surprise under sadness and training effect of disgust under neutral of females were less than those of males. The current study found that initially, the general microexpression recognition ability of females was not greater than that of males. This might be because dynamic microexpression recognition was more difficult, so that the respective advantages of males and females could not be fully utilized, while their disadvantages were masked. Females outperformed males in learning microexpression recognition techniques and made greater improvements. The training effect could detect the gender differences in the microexpression recognition learning. However, the effect sizes (η2) were too small. Therefore, the very small effect sizes might make the gender differences of small value.

Some previous research has documented a female advantage in emotion recognition that tends to remain constant across the lifespan but gradually declines with age (Brummett et al., 2008; Montagne et al., 2005; Whittle et al., 2011; Zhu et al., 2013). The current study found that there was no gender difference in the microexpression recognition ability, and females outperformed males in training effects but with too small effect size. The existing studies on microexpressions have not yielded consistent results regarding the advantage of females (Frank et al., 2009; Hurley et al., 2014; Hall and Matsumoto, 2004; Mufson and Nowicki, 1991; Yin et al., 2020). The microexpression recognition contains emotion recognition, but the emotion recognition advantage of females is uncertain to show up in microexpression recognition. It may be because the processing mechanism of microexpression recognition is different from that of macroexpression recognition. The microexpression recognition contains dynamic microexpression detection and emotion recognition. The dynamic microexpression detection encompasses expression changing and the process of the generation of perceptual consciousness (Yin et al., 2020; Zhang et al., 2020, 2022). It leads to the occurrence of emotion recognition at various levels of consciousness, such as guess (implicit), intuition, ambiguity, and seeing (explicit). This is different from the expression detection and emotion recognition of macroexpression, which occurs at a seeing level (explicit). In the future, the gender difference and its age effect, separation of expression detection and emotion recognition, and the consciousness should be compared between microexpression and macroexpression recognition.

### 4.4 Limitations, prospects and innovation

The sampling bias (sex imbalance, young people) limited the generalization of the results of the current study. It was a limitation that the sample was heavily skewed toward females (16 males, 78 females), which might lead to that the dynamic microexpression recognition accuracy and its training effect were more favorable for females. Fortunately, the 16 males reached the minimum sample size 10 events per variable (10 EPV, Mcculloch, 2007; Ogundimu et al., 2016) required for ANOVA, so the statistical analysis results for the gender differences were reasonable. Because the effect size is not affected by the sample size, the very small effect sizes (η2) of gender differences showed that even if more male participants were included, the results might not change much. Of course, this needs to be verified through future research. The use of only young university students further restricted generalizability. This was another limitation that restricted the effectiveness of DMETT to only young people. As people age, the ability to recognize microexpressions may decline (Hurley et al., 2014; Yin et al., 2016), but it is unknown whether the training effect increases, decreases, or remains unchanged. Therefore, in the future, more male participants and other age groups should be included to further test the universality of DMETT.

All participants were the yellow-skinned people of China, but they were trained to recognize dynamic microexpression of white models. This might affect the training outcome (Yin et al., 2016). However, only expression database FEEST by Young et al. (2002) met the requirements of the current study, that is, each model has 7 basic expressions, and the intensity of the expression increases progressively. Fortunately, these basic expressions have been proven to exist in many different cultures and races (Matsumoto et al., 2007). Therefore, the mechanism by which yellow-skinned people recognize the microexpressions of white people should have many commonalities with the mechanism by which yellow-skinned people recognize the microexpressions of other yellow-skinned people. This requires further research in the future.

The purpose of the current study was to establish DMETT. Therefore, based on the sample size of existing studies (Yan et al., 2013; Yin et al., 2020; Zhang et al., 2017; Zhang et al., 2020, 2022; Zhu et al., 2017; Zhu et al., 2019), valid data from 94 participants was obtained. This was inevitably unable to cover the various factors that influenced DMETT, such as gender, age, race, culture, occupation, and individual psychological characteristics (intelligence, memory, cognitive control ability, perceptual sensitivity, personality, consciousness, social skills, mental health, etc. Yin et al., 2016; Yin et al., 2020). Therefore, the scale of the current study was still relatively small. Future research should adopt diverse large sample to explore the influence of these factors on DMETT and their interaction effect.

The pretest stage used the participants’ publicly available data in Zhang et al. (2024), which was also the published research conducted by the authors of the current study. The participants continued to complete the training stage and the posttest stage in the current study. The current study focused on the training effect rather than the pretest dynamic microexpression recognition ability in Zhang et al. (2024), which was the difference and innovation. First, a dynamic microexpression recognition ability test (DMERT) was established with good reliability and validity in Zhang et al. (2024), which effectively measured the dynamic microexpression recognition ability. Then, the dynamic microexpression recognition ability was trained by DMETT in the current study. The former study was the prerequisite for the latter study. It was a logical progressive relationship. In both the current study and Zhang et al. (2024), the data of 42 subcategories and 6 categories of dynamic microexpressions and the general dynamic microexpression for each participant was availability, and the raw, trial-level data can be obtained by contacting the corresponding author. This would improve reproducibility.

Despite these limitations above, by moving from static to dynamic microexpression training, the current study addressed a key limitation of existing microexpression training tools, such as the METT (Ekman, 2002, 2003), which focus exclusively on static microexpression recognition in a neutral background. The current study developed DMETT, a novel training tool for dynamic microexpression recognition with multiple variables, including emotional background expressions, levels of emotional arousal, and types of dynamic microexpressions. The empirical evidence for effectiveness, reliability, and validity of DMETT was provided. Compared to static microexpressions, dynamic microexpressions are closer to the real ones (Ekman and Friesen, 1969; Shen et al., 2012; Takalkar et al., 2018; Yan et al., 2013; Yan et al., 2013; Yan et al., 2014; Zhang et al., 2024). Therefore, DMETT may be more capable of transferring and generalizing the improvement in dynamic microexpression recognition ability to the improvement of real microexpression recognition ability in real life, which may enhance people’s abilities in identifying lies, perceiving others’ emotions, and social skills, etc. DMETT also provides the behavioral tool for the future study of the brain mechanisms underlying the dynamic microexpression recognition ability training (Yin et al., 2016).

## 5 Conclusion

The DMETT established in the current study could effectively improve the dynamic microexpression recognition ability. The METT recognition techniques were suitable for training dynamic microexpression recognition ability. The DMETT can be used as a training tool of dynamic microexpression recognition ability, and can be further verified to serve as a measurement tool of microexpression learning ability in the future. There was no gender difference in general dynamic microexpression recognition ability, but females outperformed males in general dynamic microexpression recognition learning with small effect sizes.

## Data Availability

The original contributions presented in the study are included in the article/Supplementary material, further inquiries can be directed to the corresponding author/s.
