# Supplementary material for: The establishment of dynamic microexpression training tool
Source: Front Psychol. 2026 Jan 29;16:1676880. doi: 10.3389/fpsyg.2025.1676880 (PMC12894323; doi:10.3389/fpsyg.2025.1676880)
Supplement: Supplementary file 1 [file Table_1.docx]

APPENDIX

Table 7. Scores of 42 subcategories of dynamic microexpression in three stages

| Dynamic microexpression | Pretest phase (*M*±*SD*) | Single sample  *t*-values | Cohen’s *d* | Training phase (*M*±*SD*) | Single sample  *t*-values | Cohen’s *d* | Posttest phase (*M*±*SD*) | Single sample  *t*-values | Cohen’s *d* |
| --- | --- | --- | --- | --- | --- | --- | --- | --- | --- |
| Sadness under sadness | 0.41±0.3 | 7.77 * * * | 0.81 | 0.8±0.22 | 27.24 * * * | 2.88 | 0.8±0.24 | 26.09 * * * | 2.64 |
| Sadness under disgust | 0.18±0.22 | 0.68 |  | 0.52±0.26 | 12.67 * * * | 1.36 | 0.31±0.24 | 5.81 * * * | 0.6 |
| Sadness under fear | 0.22±0.21 | 2.42 * | 0.25 | 0.47±0.25 | 11.21 * * * | 1.21 | 0.36±0.26 | 7.34 * * * | 0.74 |
| Sadness under anger | 0.24±0.23 | 3.04 * * | 0.32 | 0.5±0.27 | 11.49 * * * | 1.23 | 0.34±0.22 | 7.6 * * * | 0.79 |
| Sadness under surprise | 0.22±0.24 | 2.25 * | 0.22 | 0.59±0.27 | 14.83 * * * | 1.57 | 0.38±0.26 | 8.15 * * * | 0.82 |
| Sadness under happiness | 0.36±0.28 | 6.63 * * * | 0.69 | 0.63±0.27 | 16.08 * * * | 1.72 | 0.5±0.28 | 11.53 * * * | 1.19 |
| Sadness under neutral | 0.31±0.29 | 4.86 * * * | 0.49 | 0.65±0.25 | 18.13 * * * | 1.93 | 0.47±0.29 | 10.07 * * * | 1.05 |
| Disgust under sadness | 0.39±0.25 | 8.58 * * * | 0.89 | 0.66±0.24 | 19.1 * * * | 2.06 | 0.61±0.26 | 16.45 * * * | 1.71 |
| Disgust under disgust | 0.43±0.24 | 10.78 * * * | 1.1 | 0.6±0.21 | 19.76 * * * | 2.06 | 0.7±0.25 | 20.99 * * * | 2.13 |
| Disgust under fear | 0.38±0.25 | 8.44 * * * | 0.85 | 0.67±0.25 | 19.23 * * * | 2.01 | 0.65±0.29 | 16.35 * * * | 1.67 |
| Disgust under anger | 0.38±0.2 | 10.63 * * * | 1.07 | 0.71±0.24 | 21.63 * * * | 2.26 | 0.54±0.24 | 14.65 * * * | 1.56 |
| Disgust under surprise | 0.41±0.3 | 7.96 * * * | 0.81 | 0.73±0.24 | 22.21 * * * | 2.35 | 0.69±0.28 | 18.02 * * * | 1.87 |
| Disgust under happiness | 0.38±0.27 | 7.65 * * * | 0.79 | 0.63±0.24 | 18.11 * * * | 1.93 | 0.58±0.26 | 15.74 * * * | 1.59 |
| Disgust under neutral | 0.54±0.29 | 12.25 * * * | 1.29 | 0.78±0.23 | 24.68 * * * | 2.67 | 0.72±0.29 | 18.78 * * * | 1.91 |
| Fear under sadness | 0.23±0.22 | 2.7 * * * | 0.29 | 0.38±0.25 | 7.97 * * * | 0.85 | 0.34±0.25 | 6.75 * * * | 0.69 |
| Fear under disgust | 0.19±0.21 | 0.95 |  | 0.43±0.23 | 10.87 * * * | 1.14 | 0.36±0.26 | 7.17 * * * | 0.74 |
| Fear under fear | 0.34±0.26 | 6.4 * * * | 0.67 | 0.65±0.25 | 18.34 * * * | 1.93 | 0.61±0.25 | 17.22 * * * | 1.77 |
| Fear under anger | 0.2±0.2 | 1.68 |  | 0.27±0.22 | 4.36 * * * | 0.47 | 0.36±0.25 | 7.3 * * * | 0.77 |
| Fear under surprise | 0.22±0.18 | 2.68 * * | 0.3 | 0.19±0.15 | 1.17 |  | 0.23±0.2 | 3.13 * * | 0.32 |
| Fear under happiness | 0.3±0.27 | 4.71 * * * | 0.49 | 0.54±0.27 | 12.84 * * * | 1.38 | 0.45±0.28 | 9.95 * * * | 1.01 |
| Fear under neutral | 0.2±0.25 | 1.49 |  | 0.35±0.27 | 6.24 * * * | 0.68 | 0.38±0.29 | 7.07 * * * | 0.74 |
| Anger under sadness | 0.2±0.2 | 1.86 * * * | 0.17 | 0.42±0.21 | 11.47 * * * | 1.21 | 0.34±0.26 | 6.29 * * * | 0.67 |
| Anger under disgust | 0.24±0.19 | 3.65 * * * | 0.39 | 0.27±0.18 | 5.41 * * * | 0.57 | 0.36±0.28 | 6.74 * * * | 0.69 |
| Anger under fear | 0.26±0.24 | 3.63 * * * | 0.39 | 0.33±0.22 | 7.1 * * * | 0.74 | 0.31±0.25 | 5.8 * * * | 0.57 |
| Anger under anger | 0.24±0.24 | 3.13 * * | 0.31 | 0.51±0.24 | 13.66 * * * | 1.43 | 0.47±0.33 | 8.94 * * * | 0.92 |
| Anger under surprise | 0.28±0.26 | 4.12 * * * | 0.44 | 0.35±0.21 | 8.18 * * * | 0.87 | 0.3±0.26 | 4.99 * * * | 0.51 |
| Anger under happiness | 0.17±0.21 | 0.22 |  | 0.32±0.19 | 7.91 * * * | 0.81 | 0.3±0.25 | 5.14 * * * | 0.53 |
| Anger under neutral | 0.23±0.23 | 2.45 * | 0.28 | 0.36±0.24 | 7.74 * * * | 0.81 | 0.27±0.3 | 3.29 * * * | 0.34 |
| Surprise under sadness | 0.63±0.32 | 14.32 * * * | 1.45 | 0.72±0.24 | 21.61 * * * | 2.31 | 0.7±0.25 | 20.88 * * * | 2.13 |
| Surprise under disgust | 0.71±0.34 | 15.32 * * * | 1.6 | 0.77±0.23 | 25.11 * * * | 2.62 | 0.73±0.27 | 20.23 * * * | 2.09 |
| Surprise under fear | 0.5±0.24 | 13.33 * * * | 1.39 | 0.54±0.22 | 16.16 * * * | 1.7 | 0.57±0.25 | 15.68 * * * | 1.61 |
| Surprise under anger | 0.66±0.35 | 13.73 * * * | 1.41 | 0.47±0.23 | 12.2 * * * | 1.32 | 0.72±0.25 | 21.49 * * * | 2.21 |
| Surprise under surprise | 0.48±0.28 | 11.11 * * * | 1.12 | 0.7±0.21 | 24.1 * * * | 2.54 | 0.75±0.24 | 24.11 * * * | 2.43 |
| Surprise under happiness | 0.63±0.33 | 13.43 * * * | 1.4 | 0.69±0.24 | 20.45 * * * | 2.18 | 0.69±0.26 | 19.64 * * * | 2.01 |
| Surprise under neutral | 0.71±0.32 | 16.67 * * * | 1.7 | 0.8±0.27 | 22.37 * * * | 2.35 | 0.78±0.29 | 20.82 * * * | 2.11 |
| Happiness under sadness | 0.67±0.33 | 14.96 * * * | 1.53 | 0.91±0.19 | 36.22 * * * | 3.91 | 0.81±0.23 | 27.33 * * * | 2.8 |
| Happiness under disgust | 0.7±0.34 | 15.16 * * * | 1.57 | 0.85±0.2 | 31.45 * * * | 3.42 | 0.83±0.23 | 27.53 * * * | 2.88 |
| Happiness under fear | 0.59±0.32 | 12.87 * * * | 1.32 | 0.8±0.21 | 28.02 * * * | 3.02 | 0.73±0.23 | 23.63 * * * | 2.45 |
| Happiness under anger | 0.73±0.34 | 15.83 * * * | 1.66 | 0.87±0.23 | 28.62 * * * | 3.06 | 0.87±0.21 | 32.73 * * * | 3.35 |
| Happiness under surprise | 0.68±0.33 | 15.03 * * * | 1.56 | 0.83±0.23 | 27.78 * * * | 2.88 | 0.83±0.21 | 31.43 * * * | 3.16 |
| Happiness under happiness | 0.45±0.34 | 8.1 * * * | 0.83 | 0.78±0.22 | 26.57 * * * | 2.79 | 0.78±0.2 | 29.93 * * * | 3.07 |
| Happiness under neutral | 0.78±0.35 | 17.19 * * * | 1.75 | 0.92±0.21 | 34.72 * * * | 3.59 | 0.91±0.22 | 32.29 * * * | 3.38 |

Note: *n*=94 for pretest and posttest, *n*=89 for training. Single sample *t* value, for 42 subcategories accuracy, the comparison standard was 1/6.

Table 8 Quantitative indicators of training effect of 42 subcategories of dynamic microexpressions

| Dynamic microexpression | TPr  (*M*±*SD*) | Single sample  *t*-values | Cohen’s *d* | PoT  (*M*±*SD*) | Single sample  *t* -values | Cohen’s *d* | PoPr  (*M*±*SD*) | Single sample  *t* -values | Cohen’s *d* |
| --- | --- | --- | --- | --- | --- | --- | --- | --- | --- |
| Sadness under sadness | 0.39±0.33 | 11.04 * * * | 1.18 | 0.01±0.2 | 0.33 |  | 0.39±0.37 | 10.36 * * * | 1.05 |
| Sadness under disgust | 0.34±0.26 | 12.38 * * * | 1.31 | -0.2±0.25 | -7.52 * * * | -0.8 | 0.13±0.25 | 4.99 * * * | 0.52 |
| Sadness under fear | 0.25±0.24 | 9.9 * * * | 1.04 | -0.11±0.27 | -3.81 * * * | -0.41 | 0.14±0.28 | 5.06 * * * | 0.5 |
| Sadness under anger | 0.26±0.26 | 9.48 * * * | 1 | -0.16±0.27 | -5.71 * * * | -0.59 | 0.1±0.26 | 3.7 * * * | 0.38 |
| Sadness under surprise | 0.37±0.27 | 12.67 * * * | 1.37 | -0.2±0.24 | -7.93 * * * | -0.83 | 0.16±0.26 | 5.98 * * * | 0.62 |
| Sadness under happiness | 0.28±0.27 | 9.57 * * * | 1.04 | -0.12±0.25 | -4.53 * * * | -0.48 | 0.14±0.29 | 4.68 * * * | 0.48 |
| Sadness under neutral | 0.34±0.33 | 9.67 * * * | 1.03 | -0.17±0.3 | -5.42 * * * | -0.57 | 0.15±0.34 | 4.4 * * * | 0.44 |
| Disgust under sadness | 0.28±0.31 | 8.61 * * * | 0.9 | -0.05±0.28 | -1.83 |  | 0.22±0.31 | 6.79 * * * | 0.71 |
| Disgust under disgust | 0.17±0.29 | 5.4 * * * | 0.59 | 0.11±0.27 | 3.78 * * * | 0.41 | 0.27±0.34 | 7.69 * * * | 0.79 |
| Disgust under fear | 0.29±0.27 | 10.33 * * * | 1.07 | -0.03±0.28 | -0.99 | -0.11 | 0.27±0.31 | 8.19 * * * | 0.87 |
| Disgust under anger | 0.32±0.28 | 11.01 * * * | 1.14 | -0.17±0.32 | -5.04 * * * | -0.53 | 0.15±0.32 | 4.54 * * * | 0.47 |
| Disgust under surprise | 0.33±0.28 | 11.24 * * * | 1.18 | -0.05±0.25 | -1.75 |  | 0.28±0.31 | 8.85 * * * | 0.9 |
| Disgust under happiness | 0.25±0.29 | 8.09 * * * | 0.86 | -0.04±0.27 | -1.29 |  | 0.2±0.31 | 6.34 * * * | 0.65 |
| Disgust under neutral | 0.24±0.35 | 6.52 * * * | 0.69 | -0.06±0.29 | -1.8 |  | 0.19±0.34 | 5.25 * * * | 0.56 |
| Fear under sadness | 0.15±0.3 | 4.83 * * * | 0.5 | -0.04±0.28 | -1.33 |  | 0.11±0.29 | 3.75 * * * | 0.38 |
| Fear under disgust | 0.25±0.25 | 9.41 * * * | 1 | -0.07±0.26 | -2.48 * | -0.27 | 0.17±0.3 | 5.53 * * * | 0.57 |
| Fear under fear | 0.32±0.32 | 9.26 * * * | 1 | -0.05±0.26 | -1.94 |  | 0.26±0.34 | 7.59 * * * | 0.76 |
| Fear under anger | 0.07±0.21 | 3.08 * * | 0.33 | 0.09±0.26 | 3.2 * * | 0.35 | 0.15±0.25 | 6.09 * * * | 0.6 |
| Fear under surprise | -0.03±0.2 | -1.21 |  | 0.05±0.24 | 2.11 * | 0.21 | 0.02±0.31 | 0.55 |  |
| Fear under happiness | 0.25±0.27 | 8.89 * * * | 0.93 | -0.09±0.32 | -2.65 * * | -0.28 | 0.15±0.36 | 4.13 * * * | 0.42 |
| Fear under neutral | 0.15±0.33 | 4.23 * * * | 0.45 | 0.04±0.32 | 1.08 |  | 0.17±0.36 | 4.57 * * * | 0.47 |
| Anger under sadness | 0.22±0.26 | 8.14 * * * | 0.85 | -0.08±0.28 | -2.65 * * | -0.29 | 0.13±0.28 | 4.54 * * * | 0.46 |
| Anger under disgust | 0.03±0.26 | 1.13 |  | 0.09±0.28 | 2.86 * * | 0.32 | 0.12±0.36 | 3.35 * * * | 0.33 |
| Anger under fear | 0.08±0.26 | 2.76 * * | 0.31 | -0.01±0.25 | -0.37 |  | 0.06±0.28 | 2.01 * | 0.21 |
| Anger under anger | 0.26±0.35 | 7 * * * | 0.74 | -0.04±0.34 | -1.23 |  | 0.22±0.44 | 4.91 * * * | 0.5 |
| Anger under surprise | 0.07±0.31 | 2.26 * | 0.23 | -0.05±0.29 | -1.58 |  | 0.03±0.35 | 0.7 |  |
| Anger under happiness | 0.15±0.25 | 5.73 * * * | 0.6 | -0.03±0.26 | -0.94 |  | 0.13±0.26 | 4.81 * * * | 0.5 |
| Anger under neutral | 0.14±0.32 | 4.09 * * * | 0.44 | -0.09±0.32 | -2.71 * * | -0.28 | 0.04±0.38 | 1.1 |  |
| Surprise under sadness | 0.1±0.28 | 3.4 * * * | 0.36 | -0.03±0.22 | -1.16 |  | 0.07±0.3 | 2.24 * | 0.23 |
| Surprise under disgust | 0.06±0.31 | 1.96 |  | -0.04±0.26 | -1.47 |  | 0.02±0.35 | 0.63 |  |
| Surprise under fear | 0.04±0.27 | 1.42 |  | 0.04±0.24 | 1.53 |  | 0.07±0.33 | 2.17 * | 0.21 |
| Surprise under anger | -0.18±0.32 | -5.15 * * * | -0.56 | 0.24±0.27 | 8.46 * * * | 0.89 | 0.06±0.36 | 1.63 |  |
| Surprise under surprise | 0.22±0.33 | 6.21 * * * | 0.67 | 0.04±0.24 | 1.65 |  | 0.27±0.35 | 7.49 * * * | 0.77 |
| Surprise under happiness | 0.07±0.29 | 2.4 * | 0.24 | 0±0.26 | -0.05 |  | 0.06±0.33 | 1.8 |  |
| Surprise under neutral | 0.1±0.33 | 2.85 * * | 0.3 | -0.03±0.28 | -0.84 |  | 0.07±0.33 | 2.19 * | 0.21 |
| Happiness under sadness | 0.24±0.3 | 7.56 * * * | 0.8 | -0.11±0.17 | -5.97 * * * | -0.65 | 0.14±0.29 | 4.52 * * * | 0.48 |
| Happiness under disgust | 0.16±0.29 | 5.12 * * * | 0.55 | -0.02±0.17 | -1.06 | -0.12 | 0.13±0.29 | 4.51 * * * | 0.45 |
| Happiness under fear | 0.21±0.29 | 6.87 * * * | 0.72 | -0.07±0.18 | -3.48 * * * | -0.39 | 0.14±0.29 | 4.8 * * * | 0.48 |
| Happiness under anger | 0.14±0.3 | 4.6 * * * | 0.47 | 0±0.18 | 0.15 |  | 0.15±0.29 | 4.92 * * * | 0.52 |
| Happiness under surprise | 0.16±0.29 | 5.18 * * * | 0.55 | 0±0.16 | 0.08 |  | 0.15±0.29 | 5.17 * * * | 0.52 |
| Happiness under happiness | 0.33±0.43 | 7.23 * * * | 0.77 | 0±0.19 | -0.21 |  | 0.33±0.4 | 7.9 * * * | 0.83 |
| Happiness under neutral | 0.15±0.32 | 4.43 * * * | 0.47 | -0.01±0.15 | -0.68 |  | 0.14±0.3 | 4.36 * * * | 0.47 |

Note: Pretest stage and posttest stage were one-to-one correspondences *n* = 94, training stage *n* = 89. TPr was the training phase minus the pretesting phase, so *n* = 89; PoT was the posttest phase minus the training phase, so *n* = 89; PoPr was posttest minus pretest, so *n* = 94. In the *t* value of a single sample, the comparison standard was 0.

Table 9 Correlation between odd and even trials of 42 subcategories of dynamic microexpressions

| PoPr | Odd | Even | *r* |
| --- | --- | --- | --- |
| Sadness under sadness | 0.37±0.43 | 0.42±0.44 | .430** |
| Sadness under disgust | 0.07±0.29 | 0.19±0.32 | .264* |
| Sadness under fear | 0.13±0.34 | 0.16±0.33 | .347** |
| Sadness under anger | 0.08±0.32 | 0.12±0.31 | .368** |
| Sadness under surprise | 0.2±0.29 | 0.13±0.36 | .298** |
| Sadness under happiness | 0.16±0.37 | 0.13±0.35 | .316** |
| Sadness under neutral | 0.15±0.43 | 0.16±0.44 | .229* |
| Disgust under sadness | 0.24±0.38 | 0.19±0.43 |  |
| Disgust under disgust | 0.3±0.4 | 0.24±0.45 | .302** |
| Disgust under fear | 0.27±0.38 | 0.26±0.35 | .466** |
| Disgust under anger | 0.11±0.44 | 0.19±0.38 | .244* |
| Disgust under surprise | 0.33±0.36 | 0.24±0.37 | .473** |
| Disgust under happiness | 0.18±0.34 | 0.23±0.39 | .450** |
| Disgust under neutral | 0.16±0.44 | 0.21±0.42 | .297** |
| Fear under sadness | 0.14±0.33 | 0.09±0.4 | .242* |
| Fear under disgust | 0.15±0.32 | 0.2±0.39 | .453** |
| Fear under fear | 0.26±0.4 | 0.27±0.42 | .343** |
| Fear under anger | 0.17±0.3 | 0.14±0.33 | .213* |
| Fear under surprise | 0.03±0.42 | 0.01±0.29 | .440** |
| Fear under happiness | 0.17±0.38 | 0.14±0.49 | .363** |
| Fear under neutral | 0.24±0.44 | 0.1±0.46 | .308** |
| Anger under sadness | 0.16±0.31 | 0.1±0.35 | .449** |
| Anger under disgust | 0.11±0.4 | 0.14±0.42 | .526** |
| Anger under fear | 0.08±0.33 | 0.03±0.36 | .346** |
| Anger under anger | 0.24±0.54 | 0.2±0.48 | .511** |
| Anger under surprise | -0.02±0.4 | 0.07±0.41 | .528** |
| Anger under happiness | 0.08±0.31 | 0.18±0.33 | .325** |
| Anger under neutral | 0.05±0.44 | 0.03±0.47 | .355** |
| Surprise under sadness | 0.07±0.43 | 0.07±0.37 |  |
| Surprise under disgust | 0.04±0.39 | 0±0.37 | .690** |
| Surprise under fear | 0.12±0.34 | 0.03±0.43 | .505** |
| Surprise under anger | 0.09±0.38 | 0.03±0.42 | .575** |
| Surprise under surprise | 0.27±0.35 | 0.26±0.44 | .537** |
| Surprise under happiness | 0.07±0.4 | 0.05±0.36 | .498** |
| Surprise under neutral | 0.1±0.39 | 0.05±0.42 | .356** |
| Happiness under sadness | 0.13±0.3 | 0.14±0.36 | .593** |
| Happiness under disgust | 0.16±0.3 | 0.1±0.35 | .562** |
| Happiness under fear | 0.14±0.29 | 0.15±0.37 | .502** |
| Happiness under anger | 0.16±0.3 | 0.13±0.33 | .737** |
| Happiness under surprise | 0.19±0.35 | 0.12±0.29 | .567** |
| Happiness under happiness | 0.35±0.46 | 0.31±0.41 | .734** |
| Happiness under neutral | 0.14±0.38 | 0.13±0.31 | .529** |

Table 10 Correlation between PoPr of 36 subcategories of dynamic microexpressions and PoPr of classic microexpressions (*r*)

| PoPr | Sadness under neutral | Disgust under neutral | Fear under neutral | Anger under neutral | Surprise under neutral | Happiness under neutral |
| --- | --- | --- | --- | --- | --- | --- |
| Sadness under sadness |  |  |  |  |  |  |
| Sadness under disgust | .347** |  |  |  |  |  |
| Sadness under fear |  |  |  |  |  |  |
| Sadness under anger | .418** |  |  |  |  |  |
| Sadness under surprise | .344** |  |  |  |  |  |
| Sadness under happiness | .258* |  |  |  |  |  |
| Disgust under sadness |  | .466** |  |  |  |  |
| Disgust under disgust |  | .390** |  |  |  |  |
| Disgust under fear |  | .345** |  |  |  |  |
| Disgust under anger |  | .242* |  |  |  |  |
| Disgust under surprise |  | .290** |  |  |  |  |
| Disgust under happiness |  | .255* |  |  |  |  |
| Fear under sadness |  |  | .473** |  |  |  |
| Fear under disgust |  |  | .452** |  |  |  |
| Fear under fear |  |  | .238* |  |  |  |
| Fear under anger |  |  | .439** |  |  |  |
| Fear under surprise |  |  | .533** |  |  |  |
| Fear under happiness |  |  | .493** |  |  |  |
| Anger under sadness |  |  |  | .338** |  |  |
| Anger under disgust |  |  |  | .506** |  |  |
| Anger under fear |  |  |  | .367** |  |  |
| Anger under anger |  |  |  | .449** |  |  |
| Anger under surprise |  |  |  | .432** |  |  |
| Anger under happiness |  |  |  | .288** |  |  |
| Surprise under sadness |  |  |  |  | .579** |  |
| Surprise under disgust |  |  |  |  | .567** |  |
| Surprise under fear |  |  |  |  | .270** |  |
| Surprise under anger |  |  |  |  | .509** |  |
| Surprise under surprise |  |  |  |  | -.203* |  |
| Surprise under happiness |  |  |  |  | .543** |  |
| Happiness under sadness |  |  |  |  |  | .558** |
| Happiness under disgust |  |  |  |  |  | .756** |
| Happiness under fear |  |  |  |  |  | .531** |
| Happiness under anger |  |  |  |  |  | .704** |
| Happiness under surprise |  |  |  |  |  | .658** |
| Happiness under happiness |  |  |  |  |  | -.354** |
